# Resistance to Systemic Agents in Renal Cell Carcinoma Predict and Overcome Genomic Strategies Adopted by Tumor

**DOI:** 10.3390/cancers11060830

**Published:** 2019-06-14

**Authors:** Veronica Mollica, Vincenzo Di Nunno, Lidia Gatto, Matteo Santoni, Marina Scarpelli, Alessia Cimadamore, Antonio Lopez-Beltran, Liang Cheng, Nicola Battelli, Rodolfo Montironi, Francesco Massari

**Affiliations:** 1 Division of Oncology, S.Orsola-Malpighi Hospital, 40138 Bologna, Italy; veronica.mollica7@gmail.com (V.M.); dinunnovincenzo88@gmail.com (V.D.N.); lidia.gatto83@gmail.com (L.G.); 2 Oncology Unit, Macerata Hospital, 62100 Macerata, Italy; mattymo@alice.it (M.S.); nicola.battelli@sanita.marche.it (N.B.); 3 Section of Pathological Anatomy, Polytechnic University of the Marche Region, School of Medicine, United Hospitals, Via Conca 71, I−60126 Ancona, Italy; m.scarpelli@univpm.it (M.S.); alessiacimadamore@gmail.com (A.C.); 4 Department of Pathology and Surgery, Faculty of Medicine, University of Córdoba, 14071 Cordoba, Spain; em1lobea@gmail.com; 5 Department of Pathology and Laboratory Medicine, Indiana University School of Medicine, Indianapolis, IN 46202, USA; liang_cheng@yahoo.com

**Keywords:** renal cell carcinoma, VEGFR, VEGF, mTOR, PD-1/PD-L1, immune-checkpoint inhibitors, target therapy, primary resistance, acquired resistance, predictive markers

## Abstract

The development of new systemic agents has led us into a “golden era” of management of metastatic renal cell carcinoma (RCC). Certainly, the approval of immune-checkpoint inhibitors and the combination of these with targeted compounds has irreversibly changed clinical scenarios. A deeper knowledge of the molecular mechanisms that correlate with tumor development and progression has made this revolution possible. In this amazing era, novel challenges are awaiting us in the clinical management of metastatic RCC. Of these, the development of reliable criteria which are able to predict tumor response to treatment or primary and acquired resistance to systemic treatments still remain an unmet clinical need. Thanks to the availability of data provided by studies evaluating genomic assessments of the disease, this goal may no longer be out of reach. In this review, we summarize current knowledge about genomic alterations related to primary and secondary resistance to target therapy and immune-checkpoint inhibitors in RCC.

## 1. Introduction

Renal cell carcinoma (RCC) represents 5% of all cancers in men and 3% in women. In 2018, it was estimated that about 65,340 new diagnoses of RCC and 14,970 RCC-related deaths occurred in United States [1]. Clear cell RCC (ccRCC) accounts for approximately 75% of kidney cancer, while the other. 25% are classified as non-clear cell renal cell carcinoma (nccRCC). In this latter subgroup, the 2016 World Health Organization recognized a broad spectrum of over a dozen histopathological entities [2]. Papillary renal cell carcinoma (pRCC) and chromophobe renal cell carcinoma (chRCC) are the most frequent subtypes (10–15% pRCC, 4–5% chRCC), while medullary, translocation and collecting duct RCC represent infrequent diagnoses. Each tumor subtype displays a specific and complex spectrum of gene mutations and molecular altered pathways resulting in a heterogeneous mixture of malignancies associated with different morphologies, immune-histochemical features, clinical behaviors and prognoses [3,4,5,6,7].

It is well known that angiogenesis is the most important hallmark of RCC. This is mainly (but not only) due to alterations of the Von Hipple Lindau (VHL) gene which occur in about 90% of ccRCC cases. Angiogenesis deregulation occurs frequently in nccRCC as well, although other genes (different from VHL) may drive this process [4,5,6].

Sunitinib, axitinib, sorafenib, pazopanib, tivozanib, cabozanitinib and bevacizumab are agents which are able to interfere and inhibit angiogenesis, mainly through the inhibition of the vascular endothelial growth factor receptor and ligand (VEGFR/VEGF). Bevacizumab is the only monoclonal antibody targeting the VEGF, while other agents inhibit the VEGFRs and other altered pathways [8,9]. Furthermore, the use of agents which are able to inhibit the mammalian target of rapamycin (mTOR) has represented a standard for metastatic RCC management [8,9,10,11,12,13,14,15,16].

Curiously, the clinical benefits of these drugs have been seen only in metastatic disease; no significant survival benefit has been demonstrated in adjuvant settings [17,18].

In the last years, a new class of compounds, immune-checkpoint inhibitors, has been evaluated in several trials for advanced/metastatic malignancies. Briefly, these agents restore immune response against tumors through the inhibition of immune-checkpoint receptors or ligands such as: programmed death receptor 1/programmed death receptor ligand 1 (PD-1/PD-L1) and cytotoxic T lymphocyte-associated protein 4 (CTLA-4). Nivolumab (anti PD-1) was the first agent approved in previously treated patients with metastatic ccRCC on the basis of the survival advantage over everolimus in a randomized phase-III trial [19,20]. In first line setting, a phase-III trial showed, for the first time, that the association between nivolumab and the CTLA-4 inhibitor ipilimumab improved the survival and other clinical outcomes of a specific population of patients with metastatic RCC [21].

Recently, two different trials investigating combination strategies (between the PD-1 inhibitor pembrolizumab and axitinib as well as the PD-L1 inhibitor avelumab and axitinib) have further. improved survival of previously untreated patients [22,23]. These studies started a third revolution, leading to a ‘’golden era’’ for the management of metastatic RCC.

Over the past few years, an increasing number of patients has benefited from these compounds. However, a large percentage of treated patients exhibit progression as best response (primary resistant patients), or progress after the achievement of an initial benefit (patients with acquired resistance). Thus, the achievement of stable and durable responses still represents a goal which is achievable only in a minority of patients. In this scenario, the evaluation of mechanisms related to resistance to treatment may be a critical issue.

Toxicity is another issue that should not be overlooked. Indeed, both TKI inhibitors, monoclonal antibody targeting VEGF and immune-checkpoint inhibitors have a specific spectrum of side effects. Toxicities strongly differ from TKI and immune-checkpoint inhibitors, but both these agents may lead to death due to side effects [24,25,26,27]. Toxicity profiles, patient individual risk (estimated through a prognostic score) and patient preferences are key elements driving the choice of systemic treatments. In this scenario, the development of predictive markers of response may avoid unnecessary toxicity in patients who would not benefit from a specific treatment.

In this review, we argue that the principal genomic alterations are associated with primary and secondary resistance to target therapy and immune-checkpoint inhibitors in RCC. Furthermore, we focus our attention on possible mechanisms related to combination strategy resistance and possible approaches to overcoming these mechanisms.

## 2. Resistance to Target Therapy

Through the administration of systemic treatments, cancer cells receive continuous external pressure, driving sub-clone selection. At the end of this process, the selection of specific clones which are refractory to treatment leads to cancer progression and ‘acquired resistance’. Acquired resistance is a process that inexorably occurs over the course of the disease. In contrast, primary resistance may occur when cancer cells do not express the specific pathways which are targetable by TKIs, or when they exhibit innate mechanisms of resistance.

Spatial (between the same tumor/metastases) and temporal (at different stages of tumor development and progression) heterogeneity are well-known behaviors of RCC, and may contribute to the development of acquired or primary resistance, not only to targeted treatments, but also to immune-checkpoint inhibitors.

### 2.1. RCC Heterogeneity and Recurrent Mutations

If genomic heterogeneity in RCC is commonly observed and leads to an extraordinary number of possible mutations, ultimately resulting in several different genomic profiles, some shared genomic alterations which have been observed in each tumor subtype act as driver mutations, providing the bases for eventual tailored approaches.

What has been suggested in RCC is that the tumor heterogeneity and genomic profile of each disease strictly depend on specific mutations occurring in different phases of disease development, with additional possible different spatial location. These mutations are often truncal-type events (e.g., chromosomal 3 losses in ccRCC) which, once they have occurred, lead to a wide spectrum of mutations.

In this model, ‘’trunks’’ mutations are represented from generally truncal-type events, while ‘’branches’’ are the possible mutations resulting from these events. The ‘’braided’’ model also hypothesizes that heterogeneous mutations may happen at different points in time, but the overall genomic profile inevitably becomes similar [28,29].

An example of this model may be observed in ccRCC. In a study of four patients, VHL mutation and 3p loss of heterozygosity were found in all regions of the tumor samples (trunk), while other common mutations recognized as driver mutations (SETD2, PBRM1, MTOR, SETD2, BAP1, KDM5C, TSC1.) were present heterogeneously (branches). These last mutations occurred in specific regions across the primary tumor or in metastatic samples, possibly reflecting a genomic response to different external pressure which may occur in different specific tumor regions [30,31].

### 2.2. Primary Resistance to Agents Targeting Angiogenesis

Regarding VEGF/VEGFR inhibitors, there are several supposed mechanisms related to primary resistance. It is important to observe that the VHL gene encodes a protein interacting with the transcription elongation factors elongin C, B (TCEB1, TCEB2) and with several other enzymatic proteins. The main task of the VHL protein is to mediate a ubiquitin-dependent degradation of the hypoxia inducible factor 1 and 2 alpha (HIF1-2α) [32]. Accumulation of HIF1-2α during hypoxia (i.e., in physiological condition) or VHL loss (pathological condition) lead to up-regulation of hypoxia-related genes such as VEGF, platelet derived-growth factor (PDGF), epidermal growth factor (EGF) and GLUT1 (the glucose transporter).

In 2008, Gordan et al. identified specific groups of patients with ccRCC who presented primary resistance to standard treatment [33]. In particular, they identified a subgroup of tumors with wild-type VHL alleles and no HIF-α expression, a subgroup with VHL deficit and HIF-1α and HIF-2α expression and tumors with VHL deficit and HIF-2α expression. The first two subgroups had an upregulation of peculiar pathways, including AKT/mTOR and extracellular-signal-regulated kinase (ERK)/mitogen-activated protein kinase (MAPK), while tumors expressing only HIF-2α presented higher c-Myc activity. Of note, this last subgroup of patients experienced primary resistance to angiogenesis inhibition. Other proposed mechanisms of primary resistance are represented by alterations occurring in specific sites of the cell membrane and in proteins related to drug transport [34]. The inhibition of apoptosis due to increased synthesis of B-cell lymphoma-2/XL (Bcl-2/XL) may be another pathway related to primary resistance [35].

### 2.3. Acquired Resistance to Agents Targeting Angiogenesis

One of the most important processes driving acquired resistance to VEGF/VEGFR inhibitors is represented by the development of other pathways driving angiogenesis. PDGF/PDGFR and mesenchymal epithelial transition receptor (MET) may be two critical pathways employed by cancer cells to overcome VEGF/VEGFR blockade [36,37,38,39,40].

As a consequence, several drugs targeting these up-regulated pathways have been evaluated in clinical trials with excellent results [41,42,43]. Fibroblast growth factor receptor (FGFR), through the FGF/FGFR pathway, drives embryonic development, thereby regulating several intracellular cascades (MAPK/ERK, PI3K/AkT, STAT, diacylglycerol protein kinase C and inositol triphosphate). Alterations of this gene have been found only in a very low percentage of RCC specimens. The activity of this gene is not fully understood, and further. evidence seems to link its hyper-expression to the development of sunitinib resistance [44,45]. However, FGFR inhibitors failed to show significant results in small clinical trials [46,47,48,49]. More recently the association between lenvatininb (a multikinase inhibitor able to inhibit also FGFR) and everolimus demonstrated very interesting activity in patients who were previously treated with RCC [50].

Interaction between the immune system and angiogenesis has gained increasing interest in recent years. In particular, the hyper-expression of IL-8 leads to VEGF mRNA transcription and autocrine VEGFR-2 activation. IL-6 activates the AKT/mTOR and STAT3 cascade, resulting in increased VEGF expression. High levels of both IL-6 and IL-8 have been associated with poor prognoses in RCC; the inhibition of the IL-6 receptor may restore angiogenesis inhibitor efficacy [51,52]. Interleukin-1α and Interleukin 1-β (IL-1α and IL-1β) may promote angiogenesis through direct stimulation of HIF-1α and VEGF transcription [53,54,55].

Metastases in tissues with high vascularization and high concentrations of blood vessels are common in RCC, and could drive primary or acquired resistance to angiogenesis inhibitors. Furthermore, circulating endothelial cells are a family of bone marrow-derived progenitors which may be responsible for angiogenesis promotion in different tumors, including RCC [53].

## 3. Resistance to mTOR Inhibitors

The phosphoinositide 3-kinase (PI3K)-AKT mammalian target of rapamycin (mTor) is a serine/threonine kinase which exists in two different complexes: the mTOR Complex 1 (mTORC1) and the mTORC2 [56].

Briefly, mTORC1 enhances protein synthesis and induces the expression of several glycolytic genes through the activation of HIF1α, while mTORC2 regulates cytoskeletal organization, thereby promoting cell-survival and metabolism [56,57]. Acquired resistance to mTOR pathways may be carried out by increased activity of the mTORC2 complex. Indeed, available mTOR inhibitors act mainly on the mTOR complex 1. As phosphorylation of mTORC2 is mediated by mTORC1, the activity of mTORC2 is enhanced by mTORC1 inhibition. This leads to increased AKT activation. Of note, GRB10 and S6K1 are two proteins which are activated by mTORC1. A lack of GRB10 and S6K1 activation results in negative feedback, thereby promoting AKT activation [58,59,60]. Phosphatase and tensin homolog (PTEN) loss seem to be associated with mTOR inhibitor activity. This occurs due to the loss of PI3K/AKT inhibition mediated by PTEN. PI3K/AKT stimulation could also result from ERK/MAPK activation, itself resulting from mTORC1 inhibition [61]. Of note, a higher percentage of reactive oxygen species may directly activate AKT pathway. The acquisition of increasing intracellular levels of reactive oxygen species is a frequent event during metabolic shift due to promoted glycolytic activity and a reduction of oxidative phosphorylation [62,63]. Thus, metabolic shift may be a mechanism of primary resistance to mTOR inhibitors.

Understanding which mechanisms are related to primary and acquired resistance is a key step toward improving treatment strategies. Indeed, the introduction of agents which are able to interact with the specific pathways which are associated with treatment resistance has improved clinical outcomes in our patients. One example is represented by cabozantinib, an effective treatment in patients who have progressed to angiogenesis inhibitors. It is probable that this success may be related to the inhibition of specific pathways, including, for example, MET receptors.

Another promising strategy is the combination of two different target agents, such as everolimus and lenvatininb. It is probable that the co-inhibition of distinct pathways may reduce the percentage of tumor cells which are refractory to one of these two drugs. This suggests that the research of combination strategies may be a promising approach to overcoming primary resistance.

## 4. Resistance to Immune-Checkpoint Inhibitors

Immune-checkpoint inhibitors represented a revolution in the management of metastatic RCC, and led to a significant improvement in patient survival. Moreover, the achievement of stable and durable responses in a small but non-negligible percentage of patients represents an additional improvement, and provides concrete hope for patients. Nonetheless, a high number of patients do not respond to these compounds, and the majority will develop progression after a variable time of response.

In Checkmate 025 (phase-III clinical trial comparing nivolumab to everolimus in patients who were previously treated with metastatic ccRCC), about 35% of patients who received nivolumab experienced progressive disease as the best response [19]. Of note, this was the first randomized trial evaluating the efficacy of an immune-checkpoint inhibitor in RCC. In the study, it was shown for the first time that immunotherapy (immune-checkpoint inhibitors) is effective for patients with metastatic disease. A combination of immune-checkpoint inhibitors or of an immune-checkpoint inhibitor and TKI has led to a higher number of responses in previously untreated patients. In Checkmate 214 (phase-III trial comparing nivolumab plus ipilimumab versus sunitinib), about 20% of patients experienced progressive disease as the best response, while about 11% experienced primary progression when treated with the combination of a PD-1 inhibitor (pembrolizumab) or PD-L1 inhibitor (avelumab) and axitinib [23,24,25]. Therefore, it seems that the inclusion of immunotherapy as part of an early line of treatment, as well as the adoption of a combination strategies, may significantly reduce the number of primary refractory patients. Nonetheless, a non-negligible percentage of patients do not benefit from combination approaches, suggesting the existence of some presently unknown molecular mechanisms of resistance. Data about primary and acquired resistance to immune-checkpoint inhibitors are mainly provided by studies investigating this issue in patients receiving these treatments alone. Moreover, several details about the various mechanisms of resistance have been obtained in patients with malignancies other than mRCC (such as melanoma). Considering these limitations, it is possible that resistance to immune-checkpoint inhibitors results from the molecular mechanisms adopted by cancer cells, and also from altered interactions among the cells which are involved in immune responses [64,65].

### 4.1. Primary Resistance to Immune-Checkpoint Inhibitors

There are several mechanisms which are thought to be related to primary resistance to immune-checkpoint inhibitors.

During tumor development, continuous external immune-pressure may increase the expression and production of interferon gamma (INFγ). Due to continuous INFγ exposure, cancers cells may develop down-regulation and/or mutations interfering with INFγ stimulation. For example, mutations occurring in the INFγ receptor chains (Janus kinases: JAK1/JAK2) or in other points of the intracellular cascade (such as the signal transducer and activators of transcription STATs) may make tumor cells refractory to CTLA-4 inhibitors [66,67].

Tumors with associated PTEN loss are usually not inflamed with a small infiltration of T-cells in the tumor contexture. PTEN loss may be associated with reduced expression of several genes, including INFγ and Granzyme B [68]. Activation of the MAPK pathways leads to increased levels of VEGF and IL-8. The latter interleukin has an inhibitory function on T-Cell activity [69]. Increased levels of β-catenin lead to the loss of a specific subset of dendritic cells (CD103), resulting in resistance to immune-checkpoint inhibitors. However, this finding has been observed only in murine models [70]. Other intracellular mechanisms related to primary resistance to immune-checkpoint inhibitors depend upon the activation of AKT and the reduction of neo-antigens production (also mediated by epigenetic modification) [65].

Immune response to tumors involves a multitude of cells with different functions. The balance between factors which are able to promote or inhibit immune responses is a key issue in understanding the mechanisms of primary and acquired resistance. Some immune-cells may inhibit and limit immune response against tumors. T regulators lymphocytes (Tregs) are an important subset of immune-cells which are able to inhibit immune response through the production of inhibitory molecules such as IL-10, transforming growth factor β (TGF-β) and IL-35 [71]. Of particular interest, increased levels of activated Tregs in tumors are associated with poor prognoses and the worst clinical outcomes. CTLA-4 inhibitors may promote a reduction of Tregs enhancing activity and the function of lymphocyte T effector (Teffs) cells, resulting in immune-response activation. In contrast, PD-1 may be a positive regulator of Tregs. As a consequence, the inhibition of PD-1 could stimulate Tregs proliferation and activation, resulting in worse clinical outcomes [64]. The correlation between PD-1 inhibitors and enhanced Tregs activity appears to be of particular interest, because it could partially explain the occurring of the hyper-progression observed in some patients treated with immunotherapy; ongoing studies evaluating the role of Tregs will give us more details about on issue. Other than Tregs, other immune cells regulate immune responses. Immune-contexture may be a critical variable in predicting clinical response/resistance to immune-checkpoint inhibitors. Recently, an extensive immune-profiling study of 74 ccRCC samples suggested that the tumor microenvironment modulates response or resistance to immune checkpoint inhibitors [72]. In this study, clinical outcomes differed significantly according to the composition of the tumor immune-associated contexture and, in particular, to the percentage of exhausted T effector cells and tumor-associated macrophages.

### 4.2. Acquired Resistance to Immune-Checkpoint Inhibitors

Acquired resistance to immune-checkpoint inhibitors also involves mechanisms occurring on primary resistance. Indeed, after a variable time of response, tumor cells could develop down-regulation or mutations in downstream cascades related to INFγ [66,67]. On the other hand, it is also possible that continuous immune response may lead to a selection of sub-clones which are associated with low or no expression of neo antigens. T-effector lymphocytes may lose their effector function, becoming unable to sustain immune responses against tumor cells [64,65]. It has also been suggested that tumor cells develop resistance through the lack of β-2-microglobulin expression. This is a protein that can promote transport of the major histocompatibility complex I (MHC Class I) on tumor cell surfaces. The lack of MHC Class I expression results in missed recognition of tumor cells by T effector lymphocytes (CD8 T Cell) [73,74]. It is well known that PD-1 and CTLA-4 are important immune checkpoints, but these are not the only pathways regulating immune response. Indeed, other immune checkpoints whose activity is still poorly understood could drive the regulation of immune response against tumors. It is possible that after a variable time of inhibition to PD-1/PD-L1 and/or CTLA-4, other immune checkpoints acquire increased activity, resulting in down regulation of immune response. Lymphocyte activation gene 3 (LAG-3, CD223) is an immune checkpoint protein whose up-regulation prevents the onset of autoimmunity. In a tumor setting, LAG-3 can lead to immunosuppression. The tumor microenvironment, with its persistent antigen exposure, leads to LAG3 overexpression. This could result in a state of immune exhaustion which is characterized by the negative regulation of T cell function. LAG3 is also expressed on activated regulatory T cells (Tregs) more than on effector T cells (Teffs) [75,76]. T cell immunoglobulin and mucin-domain containing-3 (Tim- 3) are type I trans membrane proteins that act as checkpoint inhibitors of immune response against cancer. Tumor cells, dendritic cells, endothelial cells, effectors and regulator lymphocytes could express this protein. In Teffs cells, the expression of TIM-3 is associated with exhausted phenotypes. Moreover, tumor cells expressing PD-L1 and galectin-9 by binding PD-1 and Tim-3 may down-regulate T cells function [77,78].

Other pathways regulating immune response have only been partially investigated, and may be concealing other potential mechanisms related to acquired resistance.

Considering the potential mechanisms of primary and acquired resistance, it appears reasonable that combination approaches may be a winning strategy which is able to overcome some of the aforementioned mechanisms and improve response to therapy. This is true for metastatic RCC, in which combining immune-checkpoint inhibitors with each other or with TKIs has yielded remarkable results. This is also true in other diseases in which immune-checkpoint inhibitors have been tested in combination with chemotherapy (such as lung and breast cancers) or with each other (melanoma). In general, we know that combinations may be better than immune-checkpoint inhibitors alone, but the best combinations (immunotherapy with target therapy or combination of immune-checkpoint inhibitors), and when to choose one over the other treatment strategy, remain to be determined. Moreover, due to the early stages in which this field of research is at present, we have not determined the percentage of patients experiencing acquired resistance and progression to combination treatment.

In conclusion, it is important to observe that, to date, we only have information about resistance mechanisms occurring in patients treated with immune-checkpoint inhibitors alone (not administered in combination) and in diseases different from mRCC.

**Figure 1:**
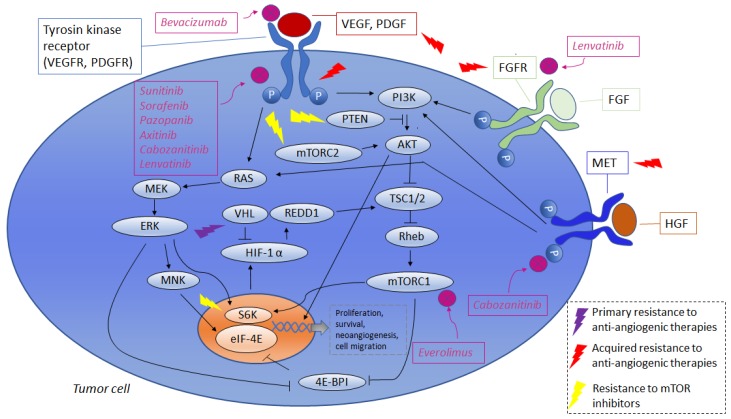
Resistance to anti-angiogenic therapies and mTOR inhibitors: VEGF or PDGF bind to a tyrosine kinase receptor and activate the PI3K and RAS pathways. PI3K activates the serine/threonine kinases AKT and mTOR. RAS activates MEK, which, in turn, activates ERK. ERK activates MNK, which, in turn, phosphorylates and activates eIF-4E. ERK and mTORC1 activate p70 S6K which, in turn, phosphorylates 4E-BP1, preventing it from binding to eIF-4E (which would cause the inactivation of the latter). The PI3K and RAS pathways result in the translation and accumulation of HIF-1α, that translocates to the nucleus and dimerizes with the constitutively-expressed HIF-1β. This complex binds to hypoxia response elements in the promoters of target genes, resulting in the transcription of genes that regulate cell growth, angiogenesis, cell survival and cell proliferation. Other pathways that may be implicated in resistance to anti-angiogenic therapies are those which are regulated by MET and FGFR. MET is activated by the binding of HGF, while FGFR is activated by FGF; this results in the activation of the RAS–MAPK and PI3K–AKT pathways, leading to the transcription of genes regulating cell proliferation, cell survival, neoangiogenesis and cell migration. Sunitinib, Sorafenib, Pazopanib, Axitinib, Cabozanitinib, Lenvatinib inhibit multiple tyrosine kinase receptors, such as VEGFR and PDGFR. Cabozanitinib inhibits MET. Lenvatinib inhibits FGFR. Everolimus inhibits mTOR. The lightning signs indicate some of the points at which resistance to therapies could arise. Cut lines indicate inhibition, and arrows indicate either. activation or induction. VEGF: Vascular endothelial growth factor; PDGF: platelet-derived growth factor; PI3K: phosphatidylinositol 3-kinase; mTOR: mammalian target of rapamycin; MEK: MAP/ERK kinase; ERK: extracellular signal-regulated kinase; MNK: MAPK-interacting protein kinase; MAPK: mitogen-activated protein kinase; eIF-4E: eukaryotic translation initiation factor 4E; S6K: S6 kinase; 4E-BP1: eIF-4E binding protein; HIF: Hypoxia-inducible factors; FGFR: fibroblast growth factor receptor; FGF: fibroblast growth factor; HGF: hepatocyte growth factor; VHL: Von Hippel-Lindau.

**Figure 2:**
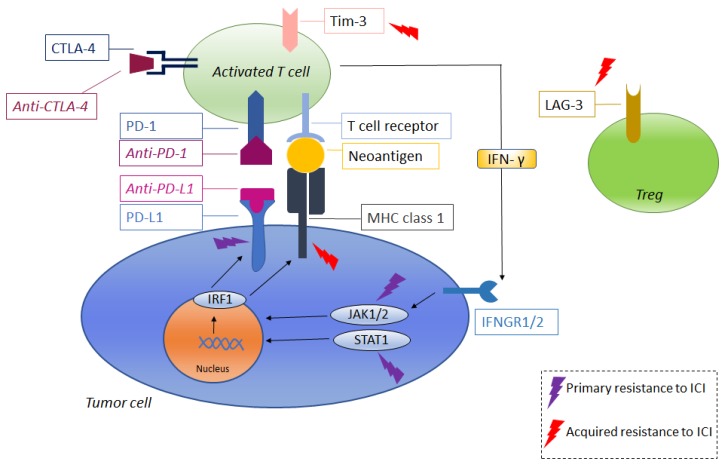
Resistance to immune-checkpoint inhibitors: Anti-PD-1 binds to PD-1 in the activated T cell, while anti-PD-L1 binds to PD-L1 in the tumor cell, thus impeding the inactivation of the immune response that would have been promoted by the tumor cell through the binding of PD-L1 to the PD-1. Anti-CTLA-4 binds to CTLA-4 in the T cell, thus impeding the downregulation of the immune system that would have originated from the binding of CTLA-4 and its ligands (B7-1 and B7-2). IFN-γ released by activated T cells binds IFNGR1/2 on tumors, activating JAK–STAT signaling that results in the activation of IFN response genes, including IRF1, which induces the transcription of other genes, leading to an increased surface expression of PD-L1 and MHC molecules. Tim-3 and LAG-3 are co-inhibitory receptors that downregulate immune response. The lightning signs indicate some of points at which a resistance to therapies could arise. Arrows indicate either activation or induction. CTLA-4: Cytotoxic T lymphocyte antigen-4; PD-1: programmed cell death-1; PD-L1: programmed death receptor ligand 1; MHC: major histocompatibility complex; TIM-3: T cell immunoglobulin and mucin-domain containing-3; LAG-3: lymphocyte-activation gene; IRF1: interferon regulatory factor 1; JAK: Janus kinase; IFN-γ: interferon-γ; IFNGR: interferon-γ receptor; Treg: regulatory T cells.

Some of the previously described mechanisms of resistance are summarized in Table 1, and are represented in Figure 1 (resistance to anti-angiogenic therapies and mTOR inhibitors) and Figure 2 (resistance to immune-checkpoint inhibitors). The percentages of patients to have experienced progressive disease as the best response (primary refractory) are reported in Table 2.

## 5. Predicting Resistance and Sensitivity to Systemic Treatment

To date, no validated approach has been able to predict the development of primary or acquired resistance to immune-checkpoint inhibitors and systemic TKIs in patients with mRCC. Before the advent of immune-checkpoint inhibitors, TKIs adopted as front-line treatments shared a common mechanism of action, i.e., one based on angiogenesis inhibition. Nowadays, the choice of treatment is performed based upon the existence of compounds with different mechanisms of action; thus, the identification of a predictive factor of response has become an urgent clinical need.

In the target therapy era, some studies have evaluated the different impact of different sequences of TKIs [78,80] on clinical outcomes, or the different outcomes (mainly patient preferences) of patients receiving alternative treatments as first line [81]. In the immune checkpoint era, the assessment of PD-L1 received particular interest, as it seemed to be a rational biomarker which was able to predict immune checkpoint inhibitor (PD-1/PD-L1 inhibitors) efficacy. In Checkmate 025, clinical benefits with nivolumab were observed, irrespective of PD-L1 expression [19]. Also as a first line, no correlation between PD-L1 expression and survival benefit was observed in patients receiving nivolumab-ipilimumab in Checkmate 214 trial (longer progression-free survival was observed in patients receiving immunotherapy with PD-L1 expression of 1% or more) [21]. PD-L1 expression seems to not assume a predictive role also when a PD-1 or PD-L1 inhibitor are combined with TKIs [22,23]. It is of interest that in checkmate 214, a significant improvement in terms of overall survival and objective response rate was observed in favor of a combination of ipilimumab and nivolumab in patients who were classified as intermediate or poor risk, according to IMDC criteria [82].

However, a non-negligible rate of complete response was also observed in good risk patients. It is probable that immune-combination may be more effective in patients with more aggressive tumors and with worse clinical behaviors; however, the factors which are able to predict responses or resistance in these categories remain to be clarified [21,22,23]. Since, to date, no predictive factors have been validated, it is probable that the choice between immune combination or immune-TKI combination strategies will be made on the basis of IMDC estimated risk, patient preference and toxicity profiles [83,84,85].

Tumor mutational burden assumes an important role, as it may predict clinical outcomes in patients with non-small cell lung cancer receiving immune-checkpoint inhibitors [86]. Even if RCC may be related to high mutational burden, the impact of this factor on predictions of response to immune-checkpoint inhibitors has not yet been fully assessed [87]. It is also probable that the detection and evaluation of immune contexture will be a critical issue which may be able to predict clinical outcomes in patients being treated with new combinations [75,88].

## 6. Conclusions

The advent of combinations of immune-checkpoint inhibitors and TKIs provides concrete hope for our patients. Other than better survival, the administration of immune-checkpoint inhibitors sometime shows complete and stable remission; thus, the word ‘cure’ may no longer be a taboo.

Unfortunately, a non-negligible percentage of patients did not respond to these treatments, while a significant number experienced progression after initial response. Understanding the mechanisms behind these occurrences is a key challenge in the development of strategies which are able to improve clinical outcomes.

Since upfront therapy will be based on immunotherapy combinations or immune-target, two important issues should be investigated in future. First, the mechanisms underlying acquired resistance in patients receiving immune-target combinations merit investigation. Indeed, the evaluation of tumor clones developing resistance to this combination represents a fundamental step in gaining an understanding of the molecular mechanisms which are related to primary and acquired resistance. Regarding combinations of the immune checkpoints, we observed that significant benefits could be obtained in patients at higher risk according to IMDC. However, some patients with a favorable risk status experienced long-term benefits from immune checkpoint combinations, while others with intermediate or poor risk statuses experienced benefits also from angiogenesis inhibitors which were adopted at time of progression to immune checkpoints. This suggests that at least two different types of RCC sub-clones exist: one which benefits from the immune checkpoint as upfront therapy, and the other which benefits from angiogenesis inhibition. The identification of these different subtypes may be important in designing personalized systemic treatments, optimizing clinical outcomes, and avoiding unnecessary toxicity.

## Figures and Tables

**Table 1:** Summary of the suggested mechanisms of acquired/primary resistance to angiogenesis inhibitors, mTOR inhibitors and immune-checkpoint inhibitors.

**Primary Resistance to Agents Targeting Angiogenesis**	
Missed expression of targets	Tumors with wild type VHL alleles without HIF-α [33]
Cellular intake of target agents	Alteration in cell-surface proteins responsible of drugs intake [34]
Apoptosis inhibition	Increased expression of (Bcl-2/XL) [35]
**Acquired Resistance to Agents Targeting Angiogenesis**	
Acquisition of novel pathways promoting angiogenesis	- PDGFR [36] - MET [37,38,39,40] - FGFR [44,45]
Interactions with immune-system	- IL-8 promotes VEGF mRNA transcription and VEGFR-2 activation [51,52] - IL-6 promotes AKT/mTOR and STAT3 cascade promoting VEGF expression [51,52]
Angiogenesis induction by interleukin	- IL-1α and IL-1β induce angiogenesis. IL-1β may stimulate production of HIF-1α and VEGF [54,55]
**Primary Resistance to mTOR Inhibitors**	
Reactive oxygen species	- Increased levels of ROS may activate AKT pathways [62,63]
**Acquired Resistance to mTOR Inhibitors**	
Increased AKT activation mediated by mTORC1 inhibition.	- Inhibition of mTORC1 leads to reduced mTORC2 phosphorylation. Increased activity of mTORC 2 resulting from reduced phosphorylation leads to AKT activation [49,50] - mTORC1 inhibition results in missed GRB10 and S6K1 activation. These proteins exert negative feedback on AKT activation [59,60] - ERK/MAPK activation may be promoted by mTORC1 inhibition and leads to PI3K/AKT activation [61,62]
**Primary Resistance to Immune-Checkpoint Inhibitors**	
Reduced response to INFγ	- Continuous INFγ exposure may lead to downregulation or mutations on pathways related to INFγ response (for example Janus kinases JAK1/2, STATs) [66,67]
Reduced expression of INFγ and other genes related to immune-response	- PTEN loss may leads to INFγ or Granzyme B reduced expression [68]
Reduced T-Cells activity	- Activation of MAPK pathways leads to increased levels of VEGF and IL-8. This last interleukin has an inhibitory function on T-Cell activity [69] - Increased levels of β-catenin may lead to the loss of a specific subset of dendritic cells (CD103) resulting in resistance to immune-checkpoint inhibitors [70]
Reduced antigen production	- Mediated by sub-clones selection and epigenetic modification [65]
Balance between cells promoting and inhibiting immune-response	- Increased proportion of Tregs leads to the production of molecules inhibiting immune-response [71] - Immune-tumor contexture enriched of exhausted T effector cells and tumor-associated macrophages [72]
**Acquired Resistance to Immune-Checkpoint Inhibitors**	
Mechanisms involved in primary resistance	- Reduced response to INFγ - Reduced expression of INFγ and other genes related to immune-response - Reduced T-Cells activity - Reduced antigen production - Balance between cells promoting and inhibiting immune-response
Reduced MHC expression	- reduced expression of beta-2-microglobulin [73,75]
Interaction of other immune-checkpoints	- LAG3 [75,76] - TIM3 [77,79]

AKT = *Protein* Kinase B, BcL 2 = B-cell lymphoma 2, FGFR = Fibroblast Growth Receptor, ERK/MAPK = extracellular signal–regulated kinases, GRB10 = Growth Factor Receptor Bound Protein 10, JAK1/2 = Janus kinases, HIF-α = Hypoxia-inducible factor 1-*alpha, LAG3 =* Lymphocyte-activation gene 3, *MET = mesenchymal*–*epithelial transition RECEPTOR, mTOR =* mammalian target of rapamycin, *PDGFR = Platelet-derived growth factor receptor, PTEN = phosphatase and tensin homolog, ROS = Reactive oxygen species, STAT3 =* signal transducer and activator of transcription 3, S6K1 = Ribosomal protein S6 kinase beta-1, *TIM-3 =* T cell immunoglobulin and mucin-domain containing-3, *VEGF = Vascular Endothelial Growth Factor, VEGFR = Vascular Endothelial Growth Factor Receptor*.

**Table 2:** Summary table reporting percentage of patients experiencing response and progressive disease (primary refractory patients) in first and second line of treatment.

Study/First Author	Year	Experimental/Comparator Arm	ORR % PD as Best Response (Tumor with Primary Resistance)
**First Line**			
NCT00098657 Motzer et al. [10]	2007	Sunitinib vs. Interferon	ORR = 31% PD = 21%
NCT00130897 Gore et al. [11]	2009	Sunitinib	ORR = 17% PD = 24%
COMPARZ Motzer et al. [14]	2013	Sunitinib vs. Pazopanib	ORR (S) = 25% PD (S) = 19% ORR (P) = 31% PD (P) = 17%
CHECKMATE 214 Motzer et al. [21]	2018	Nivolumab + Ipilimumab vs. Sunitinib	ORR (N + I) = 42% PD (N + I) = 20% ORR (S) = 27% PD (S) = 17% *
KEYNOTE 426 Rini et al. [22]	2019	Pembrolizumab + Axitinib vs. Sunitinib	ORR (P + A) = 59% PD (P + A) = 11% ORR (S) = 36% PD (S) = 17%
JAVELIN RENAL 101 Motzer et al. [23]	2019	Avelumab + Axitinib vs. Sunitinib	ORR (A + A) = 51% PD (A + A) = 11% ORR (S) = 26% PD (S) = 22%
**Second Line**			
METEOR Choueiri et al. [41]	2015	Cabozantinib vs. Everolimus	ORR (C) = 21% PD (C) = 14% ORR (E) = 5% PD (E) = 27%
CHECKMATE 025 Motzer et al. [19]	2015	Nivolumab vs. Everolimus	ORR (N) = 25% PD (N) = 35% ORR (E) = 5% PD (E) = 28%
NCT00678392 Motzer et al. [16]	2013	Axitinib vs. Sorafenib	ORR (A) = 23 PD (A) = Not reported ORR (So) = 12 PD (S) = Not reported

* Intermediate-Poor Risk patients according to IMDC. (E) = Everolimus, (S) = Sunitinib, (P) = Pazopanib, (So) = Sorafenib, (C) = Cabozantinib, (N) = Nivolumab, (A + A) = Avelumab-Axitinib, (P + A) = Pembrolizumab-Axitinib, (N + I) = Nivolumab-Ipilimumab, ORR = Objective Response Rate, PD = Progressive Disease.
